# Thomas Dover, M. B., Physician and Buccaneer

**Published:** 1899-03

**Authors:** 


### THOMAS DOVER, M.B., PHYSICIAN AND BUCCANEER.

Thomas Dover, the doctor, entered the company of posterity, and has drifted into our modern life on a powder label; while Thomas Dover, the buccaneer, third in command and one of the principal owners of the *Duke* and *Duchess* (two privateers of ancient Bristol), discoverer of Alexander Selkirk (the original Robinson Crusoe), in spite of enduring claims on our gratitude, has been forgotten.

The *Quarterly Medical Journal*, vol. vii, part I, published at length an account of the life of Dover, obtained from various sources and notably from an article by Dr. Wm. Osler.

Thomas Dover, born in 1660, was a Bachelor of Medicine of Cambridge. He settled in practice in Bristol, and 1708 he joined a group of Bristol merchants in a privateering expedition to the South Seas. The narrative of the voyage was published by Captain Woodes Rogers in "A Cruising Voyage Round the World, 1708–1711," London, 1712. This particular voyage was made memorable chiefly on account of the discovery of Robinson Crusoe on the Island of Juan Fernandez. The expedition later sacked the two cities of Guayaquil, Dover leading the van in the assault. Several ships were taken as prizes, and as captain of one of these vessels, the *Bachelor*, Dover returned with the expedition to England. As the expedition is said to have realized £170,000, Dover as a considerable owner, must have retired from buccaneering a very wealthy man. It was as late as 1731, when Dover was about seventy years of age, that he settled permanently in London. It was late in life to begin a London practice, but he quickly gained notoriety by a method even then in vogue, of writing himself into practice. His work published in 1732 was entitled "The Ancient Physician's Legacy to His Country, Being what He Has Collected Himself in Forty-nine Years of Practice." It was distinctly a popular work, purporting to describe the diseases of mankind in so plain a manner *that any person may know the nature of his own disease, together with the several remedies for each distemper faithfully set down.*

The work is said to have made a great noise in London, and was the subject of discussion in almost every coffee-house. Later editions abound in letters from grateful patients extolling his virtues. In the section on Gout is given the formula of his favorite powder: "Take opium, 1 ounce; saltpetre and tartar vitriolated, each 4 ounces; ipecacaunha, 1 ounce."

He regarded quicksilver as a specific for almost every disease, often ordering the patient an ounce daily of crude mercury. He was continually at war with his fellow physicians and the apothecaries (the general practitioners of that day), and his assaults on his associates were a constant source of amusement to the coffee-house frequenters, among whom his book made such a stir. He is described as "a man of rough temper, who could not easily agree with those about him." He died in 1741 or 1742.—*Medical Age.*

